# A Bayesian approach for analysis of ordered categorical responses subject to misclassification

**DOI:** 10.1371/journal.pone.0208433

**Published:** 2018-12-13

**Authors:** Ashley Ling, El Hamidi Hay, Samuel E. Aggrey, Romdhane Rekaya

**Affiliations:** 1 Department of Anismal and Dairy Science, University of Georgia, Athens, Georgia, United States of America; 2 USDA Agricultural Research Service, Fort Keogh Livestock and Range Research Laboratory, Miles City, Montana, United States of America; 3 Institute of Bioinformatics, University of Georgia, Athens, Georgia, United States of America; 4 Department of Poultry Science, University of Georgia, Athens, Georgia, United States of America; 5 Department of Statistics, University of Georgia, Athens, Georgia, United States of America; Southwest University, CHINA

## Abstract

Ordinal categorical responses are frequently collected in survey studies, human medicine, and animal and plant improvement programs, just to mention a few. Errors in this type of data are neither rare nor easy to detect. These errors tend to bias the inference, reduce the statistical power and ultimately the efficiency of the decision-making process. Contrarily to the binary situation where misclassification occurs between two response classes, noise in ordinal categorical data is more complex due to the increased number of categories, diversity and asymmetry of errors. Although several approaches have been presented for dealing with misclassification in binary data, only limited practical methods have been proposed to analyze noisy categorical responses. A latent variable model implemented within a Bayesian framework was proposed to analyze ordinal categorical data subject to misclassification using simulated and real datasets. The simulated scenario consisted of a discrete response with three categories and a symmetric error rate of 5% between any two classes. The real data consisted of calving ease records of beef cows. Using real and simulated data, ignoring misclassification resulted in substantial bias in the estimation of genetic parameters and reduction of the accuracy of predicted breeding values. Using our proposed approach, a significant reduction in bias and increase in accuracy ranging from 11% to 17% was observed. Furthermore, most of the misclassified observations (in the simulated data) were identified with a substantially higher probability. Similar results were observed for a scenario with asymmetric misclassification. While the extension to traits with more categories between adjacent classes is straightforward, it could be computationally costly. For traits with high heritability, the performance of the methodology would be expected to improve.

## Introduction

Error in the measurement and recording of data, especially discrete data, is a prevalent issue that has the potential to affect the results of any analysis through the reduction of the statistical power and the increase in bias [1–6]. While measurement errors can occur in continuous and categorical variables, it has been shown that their impact on inference is more severe in discrete data [7]. When error occurs in categorical variables it is commonly called misclassification and is a topic that has drawn considerable research due to its negative effect on the inference. More specifically, misclassification occurs with the assignment of a discrete response to a class that does not accurately reflect its true state [8]. For complex and heterogeneous diseases, several subclasses with varying phenotypes often exist. Using typical diagnostic technologies, the different subclasses can be difficult to differentiate for various reasons; samples may look alike under microscopic analysis, individuals may share the same symptoms or markers used in the diagnostic, or one or more subclasses of the disease may simply be unknown [5]. This similarity can consequently lead to misdiagnosis [9]. It is well documented in the literature that misclassification in a response variable will have two primary inferential implications: 1) biased parameter estimates and 2) a reduction of statistical power [1,6,10–11]. Practically, this will manifest as inaccurate estimates of the model parameters and a reduced ability to identify truly influential explanatory factors. In recent years, there has been increased discussion of a lack of reproducible research across a wide range of fields. While many factors are likely to contribute to irreproducibility, inaccurate measurements have been implicated as a potential culprit [12–14]. Error can be introduced at various stages of data collection and analysis, and it has been estimated that error rates in many datasets can be expected to fall between 1–5% if measures to prevent them are not taken [15]. For human disease data, much higher error rates have been reported. In a 2010 report by the Pennsylvania Patient Safety Authority, diagnostic errors in acute care ranged between 3.1 and 49.8% [16].

The challenges presented by misclassification are not overcome by a large sample size as might be expected [17–18]. Therefore, as “Big Data” becomes more accessible and utilized, it will be essential to consider the potential for misclassification in these datasets [19–20]. It has even been suggested that some large datasets may be more susceptible to misclassification depending on the method and/or purpose of collection, such as electronic medical records [20].

Given the negative effects of misclassification on inference, ignoring its presence during analysis would not be a reasonable approach. A variety of methods for dealing with misclassification in a response have been reported [21–23]. The majority of these methods rely on the estimation of misclassification rates (e.g. false positives and false negatives). A double sampling approach has been suggested [10,24] that estimates rates of misclassification through the use of two tests: 1) an inexpensive fallible classifier for the full sample and 2) a more expensive infallible classifier (or “gold standard”) for a partial sample. In many situations, though, a perfect or even high accuracy classifier will either be inaccessible or nonexistent. Thus, misclassification has become largely a statistical problem and several methods for addressing it have been presented over the years. Several likelihood-based methods have been suggested for dealing with misclassification [3–4, 25–29]. Furthermore, [30] presented an elegant parametric and semi-parametric approach for analyzing speaking fluency of immigrants that contemplates errors due to subjective evaluations and scaling across individuals. Although these likelihood-based approaches are able to handle the misclassification problem, they seem not to perform well when the misclassification probabilities are small.

Advances in Markov Chain Monte Carlo (MCMC) simulation techniques have greatly facilitated the use of Bayesian methods in misclassification problems. Hierarchical Bayesian modeling with conjugate informative priors has become a method of choice for analysis of misclassified binary data. Several studies by our group [5–6,11] and others [31–32] have been successful in dealing with misclassification errors in binary data under various sampling models. However, limited research has been carried out to scale those methods to the multinomial response situation. In this study, the methods presented by our group to deal with misclassified binary traits will be extended to multinomial response situations using a hierarchical Bayesian framework. Although our proposed approach was developed and implemented before [33] published their paper on dealing with unidirectional misclassification of ordinal covariates, both methods overlap on their approach to the problem. However, our method is unique as it addresses misclassification on response variables and it allows for asymmetric misclassification probabilities within a full Bayesian implementation.

## Materials and methods

Let ***y*** *= (y*_*1*_, *y*_*2*_,*…*, *y*_*n*_*)’* be a vector of ordered categorical responses collected on *n* individuals with *y*_*i*_ taking the value of one of *C* mutually exclusive discrete responses. This observed data is considered to be a noisy representation of true unobserved data, ***r*** *= (r*_*1*_, *r*_*2*_,*…*, *r*_*n*_*)’*. The distribution of *y*_*i*,_ conditional on the probability of observing each response, is given by

$$p\left(y_{i}|\theta_{i}^{*}\right)=\Pi_{k=0}^{C-1}{\left(\theta_{ik}^{*}\right)}^{I\left(y_{i}=k\right)},$$ [1]

where θ^*^_i_ = (θ^*^_i0_, θ^*^_i1_,*…*, θ^*^_i(C-1)_*)’* is the vector of probabilities that observation *y*_*i*_ for individual *i* is observed as class *k*, *p(y*_*i*_ *= k)*, taking a value for each of the *C* discrete classes of the noisy data; and *I(y*_*i*_ *= k)* is an indicator function that takes the value of one if *(y*_*i*_ *= k)*, otherwise equaling zero.

Obviously, the observed probabilities of the multinomial distribution in [1] are linear combinations of the true probabilities, *θ*_*ij*_, and the misclassification probabilities, *π*_*jk*_, which is the probability that an observation with true class *j* is classified as observed class *k*. Misclassification will occur when *j* does not equal *k*. Thus, for class *k*, θ^*^_ik_ could be presented as

$$\theta_{ik}^{*}=\pi_{0k}\theta_{i0}+\pi_{1k}\theta_{i1}+\ldots +\pi_{kk}\theta_{ik}+\ldots +\pi_{\left(c-1\right)k}\theta_{i\left(c-1\right)},$$ [2]

where θ^*^_ik_ is the probability of observing class *k* for individual *i*, *θ*_*ij*_ is the true probability of observing the discrete response class *j* for individual *i*, and *π*_*jk*_ is the conditional probability of observing class *k* when the true class is *j*. When *j* is different from *k*, *π*_*jk*_ is the misclassification probability from true class *j* to observed class *k*. Thus,

$$\theta_{ik}^{*}=\left[\pi_{0k}\;\pi_{1k}\ldots \pi_{kk}\ldots \pi_{\left(c-1\right)k}\right]\theta_{i}=\pi_{k}\theta_{i}.$$ [3]

In matrix notation and generalizing to all elements of the vector, θ^*^_i_, Eqs [2 and 3] can be re-written as

$$\theta_{i}^{*}=\left[\begin{array}{ccc} \pi_{00} & \cdots & \pi_{\left(c-1\right)0}\\ \vdots & \ddots & \vdots \\ \pi_{0\left(c-1\right)} & \cdots & \pi_{\left(c-1\right)\left(c-1\right)} \end{array}\right]\theta_{i}=\left[\begin{array}{c} \pi_{0}\\ \vdots \\ \pi_{\left(c-1\right)} \end{array}\right]\theta_{i}=P\theta_{i},$$ [4]

where the matrix ***P*** assembles the conditional probabilities, *π*_*jk*_, between all classes. The vectors θ^*^_i_ and ***θ***_***i***_ are specific to each individual and they could be modeled as a function of systematic and random effects. However, the matrix ***P*** is common to all individuals. The matrix ***P*** does not have to be symmetric and the only requirement is that columns sum to 1.

In the absence of misclassification, the matrix ***P*** will be equal to the identity matrix, as expected, and θ^*^_i_ **= *θ***_***i***_.

Assuming conditional independence, the joint distribution of the true data, ***r***, conditional on the vector of true probability, ***θ***_***i***_ = (*θ*_*i0*_, *θ*_*i1*_,…, *θ*_*i(C-1)*_)’ is given by

$$p\left(r|\theta \right)=\Pi_{i=1}^{n}\Pi_{j=0}^{C-1}{\left(\theta_{ij}\right)}^{I\left(r_{i}=j\right)},$$ [5]

where *I(r*_*i*_ *= j)* is an indicator function that takes the value of one if *(r*_*i*_ *= j)* and otherwise is equal to zero.

Let ***α*** *= (α*_*1*_, *α*_*2*_, *…*, *α*_*n*_*)’*, where *α*_*i*_ is an unknown random variable indicating if the observed discrete response for individual *i* arose from a switching (misclassification) event. Assuming that the observed (and potentially misclassified) discrete response of individual *i* is *k* (*k* ϵ {0,1,…,C-1}), then α_i_ can be modeled following a multinomial process with probability vector ***π***_***k***_ *= (π*_*0k*,_ *π*_*1k*,_ *… π*_*jk*,_ *… π*_*(C-1)k*_*)*,

$$p\left(\alpha |P\right)=\Pi_{i=1}^{n}\Pi_{j=0}^{C-1}{\left(\pi_{jk}\right)}^{I\left(\alpha_{i}=h\right)},$$ [6]

where *h* is an index taking *C* discrete values and *I(α*_*i*_ *= h)* is an indicator function that takes the value of one if *(α*_*i*_ *= h)* and otherwise is equal to zero.

The joint distribution of ***α*** and ***r*** given ***θ*** and ***π*** can be written as

$$p\left(r,\alpha |\theta ,P\right)=\Pi_{i=1}^{n}\Pi_{j=0}^{C-1}{\left(\pi_{jk}\right)}^{I\left(\alpha_{i}=h\right)}{\left(\theta_{ij}\right)}^{I\left(r_{i}=j\right)}.$$ [7]

The joint distribution in Eq [7] depends on the true discrete data, ***r***, which is not available. However, such data could be generated as a function of the observed contaminated discrete responses, ***y*,** and the vector ***α***. Let *f()* be a link function that relates the true discrete data to the noisy responses and the vector of indicator variables ***α***

$$r_{i}=f\left(y_{i},\alpha_{i}\right).$$ [8]

For example, if the observed and potentially noisy discrete response for individual *i* is *y*_*i*_ = 0 then the true unobserved response, *r*_*i*_, can be generated using the relationship

$$r_{i}=\left(1-\alpha_{i}\right)y_{i}+\alpha_{i}\left(1-y_{i}\right),$$

with *α*_*i*_ taking the value of 0 (no misclassification), 1 (switching from *r*_*i*_ = 1 to *y*_*i*_ = 0) and 2 (switching from *r*_*i*_ = 2 to *y*_*i*_ = 0). A similar relationship could be built when the observed discrete response *y*_*i*_ is different from zero.

Using the relationship in Eq [8], the joint distribution in Eq [7] could be re-written as a function of the observed data,

$$p\left(y,\alpha |\theta ,P\right)=\Pi_{i=1}^{n}\Pi_{j=0}^{C-1}{\left(\pi_{jk}\right)}^{I\left(\alpha_{i}=h\right)}{\left(\theta_{ij}\right)}^{I\left(f\left(y_{i},\alpha_{i}\right)=j\right)},$$ [9]

where *I*(*f*(*y*_*i*_, *α*_*i*_) *= j*) is an indicator function that takes the value of one if (*f*(*y*_*i*_, *α*_*i*_) *= j*) and otherwise is equal to zero.

It is often the case that the probability of observing a specific outcome of the multinomial process, *θ*_*ij*_, is a function of a set of systematic (***β***) and random (***u***) effects. Thus, from hereafter *θ*_*ij*_ will be denoted by *θ*_*ij*_(***β***, ***u***) to indicate this relationship.

To finalize the Bayesian formulation, prior distributions are needed for the unknown parameters in the model. For the systematic and random effects the following priors were specified:

$$p\left(\beta \text{|}\beta_{min},\beta_{max}\right])\sim U\left[\beta_{min},\beta_{max}\right],$$ [10]

$$p\left(u\text{|}A,\sigma_{u}^{2}\right)\sim N\left(0,A\sigma_{u}^{2}\right).$$ [11]

where ***β***_*min*_ and ***β***_*max*_ are known hyperparameters, ***A*** is a known symmetric covariance matrix between the elements of the vector ***u***, and *σ*_*u*_^*2*^ is an unknown dispersion component for which a scaled inverse chi-squared with *v* degrees of belief and scaling factor *s*^*2*^ was used as the prior.

$$p\left(\sigma_{u}^{2}\text{|}\nu ,s^{2}\right)\sim \chi^{-2}\left(\nu ,s^{2}\right)$$ [12]

***β***_*min*_ and ***β***_*max*_ are hyperparameters to be specified by the researcher based on published estimates in the literature, based on the researcher’s own experience of the range of values the parameter might take, or by choosing an interval large enough to contain all reasonable values of the parameter.

For the misclassification probabilities vector, ***π***_***k***_, a Dirichlet distribution was assumed,

$$p\left(\pi_{k}\text{|}\tau \right)=\frac{1}{B\left(\tau_{k}\right)}\Pi_{j=0}^{C-1}\pi_{jk}^{\tau_{jk}-1}=Di\left(\pi_{k};\;\tau_{k}\right),$$ [13]

where ***τ*** = (τ_0k_, τ_1k_, …, τ_(c-1)k_) > 0 is the vector of concentration parameters of the Dirichlet distribution, B(***τ***_***j***_) is the multivariate Beta function that plays the role of a normalizing constant, and *π*_*jk*_ is the probability of misclassification (switching) from discrete response class *j* to class *k* as defined previously in Eqs [2] and [3].

The joint posterior distribution of all unknowns in the models is proportional to the product of the expressions in Eqs [9–13],

p(β,u,α,P|y)∝

$$\Pi_{i=1}^{n}\Pi_{j=0}^{C-1}{\left(\pi_{jk}\right)}^{I\left(\alpha_{i}=h\right)}{\left[\theta_{ik}\left(\beta ,u\right)\right]}^{I\left(f\left(y_{i},\alpha_{i}\right)=j\right)}.\left(\sigma_{u}^{-\frac{q+\nu }{2}}\right)\exp\left(-.5\frac{\left(u^{\prime }A^{-1}u+s^{2}\right)}{\sigma_{u}^{2}}\right).\Pi_{k=0}^{C-1}\frac{1}{B\left(\tau_{k}\right)}\Pi_{j=0}^{C-1}\pi_{jk}^{\tau_{jk}-1}\;for\;\beta_{min}\leq \beta \leq \beta_{max},$$ [14]

where *q* is the number of elements in the vector ***u***. In genetics studies, *q* will be equal to the number of individuals in the pedigree file, a file listing the ID of each animal in the population along with its sire and dam ID (where a zero is used for unknown sires and dams). Inclusion of pedigree information in the model is essential as it relates information concerning genetic merit of an individual through its relatives and information on the relatives.

A data augmentation algorithm as described by [34] and [5] will facilitate the implementation of the model in Eq [14]. It consists of assuming the existence of an unknown continuous random variable, *l*_*i*_, that relates to the discrete response via the following relationship:

$$p(y_{i}=k\text{|}l_{i},t_{k-1},t_{k}\text{)}=\{\begin{array}{c} 1\;\;\;\;if\;t_{k-1}<l_{i}\leq t_{k}\\ 0\;\;\;\;\;\;\;\;\;\;\;\;\;\text{otherwise} \end{array},$$

where *t*_*j*_ is an arbitrary threshold value. If *y*_*i*_ can take a value in one of *C* mutually exclusive ordered categorical responses, then there will be *C*+1 thresholds *(t*_*-1*_, *t*_*0*_, *t*_*1*_, *…*, *t*_*C-1*_*)* where *t*_*-1*_ and *t*_*C-1*_ are often set equal to negative and positive infinity.

The underlying liability for individual *i* could be modeled as a function of the systematic and random effects,

$$l_{i}=x_{i}\beta +z_{i}u+e_{i},$$

Where ***x***_***i***_ and ***z***_***i***_ are known incidence matrices relating the liability of individual *i* to the set of systematic and random effects, respectively, and *e*_*i*_ is the error term. Augmenting the model in Eq [7] with the vector of liabilities results in a lack of identifiability. To make the augmented model identifiable [34], two restrictions are needed. It was assumed that *t*_*0*_ = 0 and *var*(*e*_*i*_) = 1.

At the liability scale, the full conditional distributions of ***β*, *u*** and *σ*_*u*_^*2*^ are identical to those obtained in a standard analysis of discrete data in the absence of misclassification and can be found in [34], [5] and [35]. These conditional distributions, needed for a Bayesian implementation of the model via the Gibbs sampler, were in closed form, and were truncated normal for the liabilities, normal for the position parameters, and scaled inverse chi-squared for *σ*_*u*_^*2*^.

The conditional of *α*_*i*_ is a multinomial distribution,

$$p\left(\alpha_{i}|\beta ,u,\alpha_{-i},P,\sigma_{u}^{2},y\right)\propto \Pi_{k=0}^{C-1}{\left(\pi_{jk}\right)}^{I\left(\alpha_{i}=h\right)}{\left[\theta_{ij}\left(\beta ,u\right)\right]}^{I\left(f\left(y_{i},\alpha_{i}\right)=k\right)},$$

where ***α***_***-i***_ is the vector ***α*** without the *i*^*th*^ position.

For the misclassification probabilities, ***π***_***j***_, its conditional distribution is proportional to

$$p\left(\pi_{k}|\beta ,u,\alpha ,P_{-k},\sigma_{u}^{2},y\right)\propto \Pi_{k=0}^{C-1}\frac{1}{B\left(\tau_{k}\right)}\Pi_{j=0}^{C-1}\pi_{jk}^{\tau_{jk}-1}\Pi_{i=1}^{n_{k}}\Pi_{j=0}^{C-1}{\left(\pi_{jk}\right)}^{I\left(\alpha_{i}=h\right)},$$

where ***P***_***-k***_ is the matrix ***P*** without the k^th^ row and *n*_*k*_ is the number of individuals with an observed discrete response equal to the k^th^ category (class).

$$p\left(\pi_{k}|\beta ,u,\alpha ,P_{-k},\sigma_{u}^{2},y\right)\propto \Pi_{k=0}^{C-1}\frac{1}{B\left(\tau_{k}\right)}\Pi_{j=0}^{C-1}\pi_{jk}^{\tau_{jk}-1}\Pi_{j=0}^{C-1}{\left(\pi_{jk}\right)}^{\gamma_{jk}}$$

$$\propto \frac{1}{B\left(\tau_{k}\right)}\Pi_{j=0}^{C-1}\pi_{jk}^{\left(\tau_{jk}+\gamma_{jk}-1\right)}.$$

where *γ*_*jk*_ is the number of discrete responses switched (misclassified) from true class *j* to the observed class *k*. Thus, ***π***_***k***_ is distributed as *Di*(***π***_***k***_; ***τ***_***k***_ + ***γ***_***k***_) with ***γ***_***k***_ = (*γ*_*0k*,_ *γ*_*1k*, *…*,_ *γ*_*(C-1)k*_). The vector ***γ***_***k***_ is easily obtained as a by-product of the sampling process.

### Data

Calving ease, a measure of how easily a cow delivers her calf, is an important trait in beef cattle production. Selection on animals with a high growth rate is of importance to the industry for the sake of yielding a higher carcass weight per animal. However, growth rate and birth weight are positively correlated traits, and so selection on a higher growth rate indirectly selects for higher birth weights. It has been found that high birth weight is the largest contributing factor in rates of dystocia, or calving difficulty, an issue that leads to several health and economic concerns, such as increased mortality for calf and cow and additional veterinary costs. It is therefore of interest for producers to consider calving ease directly as a trait in their selection models. However, without strict protocols on the farm, measuring calving ease may involve a significant degree of subjectivity.

This study was largely motivated by a real beef cattle dataset. The data consisted of calving ease and related measurements in a composite breed of beef cattle obtained from the USDA Fort Keogh Range and Research Laboratory in Miles City, Montana [36–37]. After quality control, the USDA data included 955 calving ease phenotypes (or observations). Calving ease was scored on a four-category scale in the raw data, with a score of 1 for the easiest calving and 4 for the most problematic (Caesarian section required); however, category 4 contained few observations and so categories 3 and 4 were combined into a single bin to avoid an extreme-case problem (ECP), when the discrete response is the same for all individuals within a given systematic effect class. The three resulting categories were scored as 0 for no difficulty, 1 for moderate difficulty and 2 for severe difficulty. The distribution of calving ease scores showed an unexpected pattern of few calvings for category 1 (moderate difficulty). Given the subjectivity of the trait (scoring carried out by farm employees) and the fact that some cows calve overnight under standard ranch conditions with limited presence or absence of ranch employees, it was reasonable to assume that the collected data could be subject to potential misclassification. In order to test the adequacy of the proposed method to deal with noisy ordered categorical data and to evaluate its robustness in the presence of a small sample size, two datasets of large and small (**D1** and **D2**, respectively) sample size were simulated.

#### Simulated data

Both datasets (**D1** and **D2**) were simulated following the structure of a real calving ease dataset. For both datasets (**D1** and **D2**), a discrete trait with three categories (*C* = 3), similar to calving ease, was simulated. The number of animals with phenotype (*n*) ranged between 8,939 and 9,042 for **D1** and 1,393 and 1,425 for **D2**. The pedigree files included 10,000 and 1,563 animals for **D1** and **D2**, respectively. A mixed linear model that includes three systematic effects and one random effect in addition to the error terms was used to model the liabilities,

l=Xβ+Zu+e,

where ***l*** is a vector of liabilities of size *n*, ***β*** and ***u*** are vectors of fixed and random (animal) effects, respectively, ***e*** is a vector of residuals, and ***X*** and ***Z*** are known incidence matrices with the appropriate dimensions.

In this model, the random effect accounts for the animal’s additive genetic contribution to the trait and is commonly called the animal additive effect. The genetic covariance between animals is accounted for through the average relationship matrix, commonly denoted as ***A***, which is computed based on the known pedigree information. The element *a*_*ij*_ of the matrix ***A*** is simply the expected additive relationship between animals *i* and *j*. A detailed algorithm for constructing the relationship matrix can be found in [38]. Solving the mixed model equations requires inversion of the relationship matrix. While the relationship matrix is relatively sparse, it’s much more computationally efficient to directly build the inverse of the relationship matrix, ***A***^***-1***^ using simple rules derived in [39].

Each systematic effect was drawn from a normal distribution. The number of levels and distributional parameters of each systematic effect are summarized in Table 1. The random effects and residual terms were generated from the following distributions:

$$u\;∼\;N\left(0,A\sigma_{u}^{2}\right),$$

$$e\;∼\;N\left(0,\;I\sigma_{e}^{2}\right),$$

where ***A*** is a known matrix of expected additive relationships between animals computed based on the information in the pedigree files [39] and *σ*_*u*_^*2*^ and *σ*_*e*_^*2*^ were set equal to 0.1 and 1, respectively, resulting in a heritability of 0.091.

**Table 1:** Number of classes and distributions used to simulate the systematic effects for the large (D1) and small (D2) datasets.

Fixed Effect	Number of Levels		Distribution
	D1	D2	
1	20	5	*N(-0*.*7*,*0*.*05)*
2	10	5	*N(-0*.*1*,*0*.*5)*
3	5	5	*N(0*.*1*,*0*.*2)*

The simulated liability was discretized to generate an ordered categorical trait with three categories (*C* = 3) using the following relationship:

$$y_{i}=\{\begin{array}{c} 0\\ 1\\ 2 \end{array}\;\;\begin{array}{c} t_{min}<l_{i}\leq t_{0}\\ t_{0}<l_{i}\leq \;t_{1}\\ t_{1}<l_{i}\leq t_{max} \end{array},$$

where *y*_*i*_ is the discrete response for animal *i*, taking the value of 0, 1, or 2; and ***t*** = (*t*_*min*_ = -∞, *t*_*0*_, *t*_*1*_, *t*_*max*_ = ∞) is a vector of threshold values with *t*_*0*_ and *t*_*1*_ arbitrarily set to 0 and 1, respectively.

Incidence rates of the three categories (0, 1, and 2) were on average (0.39, 0.38, and 0.23) and (0.36, 0.46, and 0.18) for **D1** and **D2**, respectively. All scenarios were replicated 10 times and ECPs were avoided.

Misclassification was introduced to the simulated data by switching a subset of observations from true class *y*_*i*_ = *j* to observed class *y*_*i*_ = *k* for (*j*, *k*) ϵ (0, 1, 2) and *j* ≠ *k*. Two scenarios of misclassification were investigated, symmetrical misclassification, where the rate of misclassification (*π*_*jk*_) between two classes is equal in both directions (*π*_*jk*_ = *π*_*kj*_), and non-symmetrical misclassification, where the rate of misclassification from class *j* to class *k* is allowed to differ from class *k* to class *j* (*π*_*jk*_ ≠ *π*_*kj*_).

For the symmetrical misclassification, a misclassification rate of 2.5% for each direction was introduced to the data (*π*_*jk*_ = 0.025 for all *j* ≠ *k*). This approach yielded a non-differential net rate of misclassification in the simulated data of 5%. For the nonsymmetrical scenario, a misclassification rate was set equal to 1, 3, 1.5, 1, 0.1, and 0.1% for *π*_*12*_, *π*_*21*_, *π*_*23*_, *π*_*32*_, *π*_*13*_, and *π*_*31*_, respectively.

#### Real data

The real data consisted of calving ease records from 955 first parity cows from a Composite Gene Combination breed (CGC; 50% Red Angus, 25% Charolais, 25% Tarentaise; [36–37]) born between 2002 and 2011 at USDA-ARS, Fort Keogh Livestock and Range Research Laboratory, Miles City, MT. The pedigree file was comprised of 1,357 animals, including 82 sires and 651 dams. Calving ease was scored on a discrete scale with three categories (0 = no difficulties; 1 = moderate difficulties; and 2 = severe difficulties), where the most severe category was composed of two separate classes that were collapsed together. The systematic effects consisted of sex (2 classes), feed treatment (2 classes) and year of birth (10 classes).

### Data analysis

Each of the simulated data sets for the symmetric scenario was analyzed using the following models: true simulated discrete data analyzed with a classical threshold model (M1), noisy simulated data analyzed with a classical threshold model that does not contemplate misclassification (M2), noisy simulated data analyzed with a threshold model that contemplates misclassification following our proposed method and misclassification probabilities assumed known (M3), noisy simulated data analyzed with a threshold model that contemplates misclassification following our proposed method and misclassification probabilities assumed unknown (M4). An additional analysis was carried out to test the validity of our approach under a null model (when no misclassification is present in the data). For that purpose, the true simulated data was analyzed with a threshold model that contemplates misclassification following our proposed method and misclassification probabilities assumed unknown (M5). The simulated data generated under the non-symmetric scenario was analyzed only with M4. Similarly, the real data was analyzed only with M2.

Implementation was carried out using the Gibbs sampler. A unique chain of 200,000 samples was implemented with the first 100,000 iterations discarded as burn-in period. The probability of each observation being misclassified was calculated as the ratio between the number of times the observation was found to be misclassified and the total number of iterations.

## Results

Under the symmetric scenario, the estimate of the genetic variance, for the large simulated data and in the absence of misclassification in the discrete response (M1), was virtually equal to the true value used in the simulation (0.10). Furthermore, the 95% highest posterior density (HPD) interval is well-centered around the true value, indicating a reduction of bias (Table 2). However, when 5% of the discrete responses were artificially misclassified but no measures to address misclassification taken during the analysis (M2), the genetic variance was grossly underestimated with a posterior mean of roughly half the true value used in the simulation (0.052). Additionally, the 95% HPD interval did not include the true genetic variance (0.10), indicating significant bias in the estimate of the parameter (Table 2). This result demonstrates that when appropriate measures are not taken to correct or account for misclassification, there will be substantial bias in parameter estimation even with low to moderate noise in the input data.

**Table 2:** Posterior means, posterior standard deviations and the 95% highest posterior density interval (HPD) of the genetic variance (true value = 0.1) under different models^1^ and datasets^2^.

	Mean		Standard Deviation		95% HPD Interval	
	D1	D2	D1	D2	D1	D2
M1	0.106	0.135	0.0255	0.0670	0.0586–0.170	0.0469–0.319
M2	0.0521	0.0919	0.0143	0.0422	0.0288–0.0846	0.0364–0.198
M3	0.112	0.151	0.0355	0.0881	0.0540–0.191	0.0460–0.380
M4	0.0998	0.106	0.0188	0.0239	0.0688–0.142	0.0681–0.161
M5	0.113	0.108	0.0226	0.0251	0.0755–0.163	0.0692–0.167

^1^ M1: True data analysis with a classical threshold model, M2: Noisy data analyzed with a classical threshold model, M3: noisy data analyzed with the proposed method assuming the misclassification probability is known, M4: same as M3 except the misclassification probability is assumed unknown, M5: Noise free data analyzed using our proposed method (null model);

^2^ D1: large dataset, D2: small dataset

When the same noisy data used in M2 was analyzed using the proposed methodology that accounts for potential misclassification, bias in estimates of genetic variance was significantly reduced. This was true both when the probability of misclassification, *π*_*jk*_, between each pair of classes *j* (*j = 1*,*2*,*3*) and *k* (*k = 1*,*2*,*3*), was assumed known and equal to the true value used in the simulation (0.025) (M3) and when it was assumed unknown and estimated during the analysis (M4). In fact, the posterior mean of genetic variance was 0.112, and 0.0998, for M3, M4, respectively. In each of these cases, the true value of the parameter (0.10) was well within the 95% HPD interval, indicating a reduction of bias.

For the non-symmetric simulation scenario, the posterior mean (SD) of the genetic variance was equal to 0.118 (0.0196) and 0.103 (0.0243) for **D1** and **D2**, respectively. These estimates are very similar to the true value used in the simulation (0.10) and based on the 95% HPD interval (information not presented), these estimates have minimal bias.

Estimates of the probabilities of misclassification under model M4 for the symmetric and non-symmetric simulation scenarios are presented in Table 3. For the symmetric scenario, the misclassification probabilities between categories 1 and 2 (*π*_12_) and 2 and 3 (*π*_23_) of the discrete responses were underestimated with posterior means of 0.0146 and 0.0135, respectively, compared to the true value of 0.025. However, the misclassification probability between categories 2 and 3 (*π*_23_) was estimated with minimal bias and the posterior mean (0.0239) is very similar to the true value (0.025). In spite of the underestimation of some of the misclassification probabilities, the results clearly indicate the ability of the proposed methodology to adjust for the misclassification in the data whether or not the true rate of misclassification is known. Similar performance was observed using data from the non-symmetric simulation scenario. In fact, five out of the six misclassification probabilities were accurately estimated and only the misclassification probability between categories 2 and 1 (*π*_*21*_) was under estimated (Table 3).

**Table 3:** Posterior mean of the misclassification probability between the different categories of the discrete responses and datasets^1^.

	Symmetrical Misclassification				
	True Value	M4		M5	
Parameter		D1	D2	D1	D2
*π*_*12*_	0.025	0.0146	0.0128	0.0112	0.0124
*π*_*23*_	0.025	0.0135	0.0062	0.0065	0.0057
*π*_*13*_	0.025	0.0239	0.0067	0.0019	0.0029
	Nonsymmetrical Misclassification				
	True Value	M4			
		D1		D2	
*π*_*12*_	0.01	0.010		0.0095	
*π*_*21*_	0.03	0.013		0.0100	
*π*_*23*_	0.015	0.011		0.0098	
*π*_*32*_	0.01	0.011		0.0099	
*π*_*13*_	0.001	0.0013		0.0013	
*π*_*31*_	0.001	0.0014		0.0011	

^1^ D1: large dataset, D2: small dataset; *π*_*ij*_ is the misclassification probability between categories *i* and *j*

To further evaluate the adequacy of the proposed methodology, we tested its performance under a null model (no misclassification in the data) under the symmetric simulation scenario. Thus, when the noise free data used in M1 was reanalyzed using our proposed model that contemplates potential misclassification (M5), the estimate of the genetic variance (0.113) was similar to the true value and minimal bias was observed (Table 2). Most importantly, the estimated misclassification probabilities were negligible, indicating that very few observations were detected as potentially misclassified (Table 3). This was expected given that the data was free of noise.

**Fig 1:**
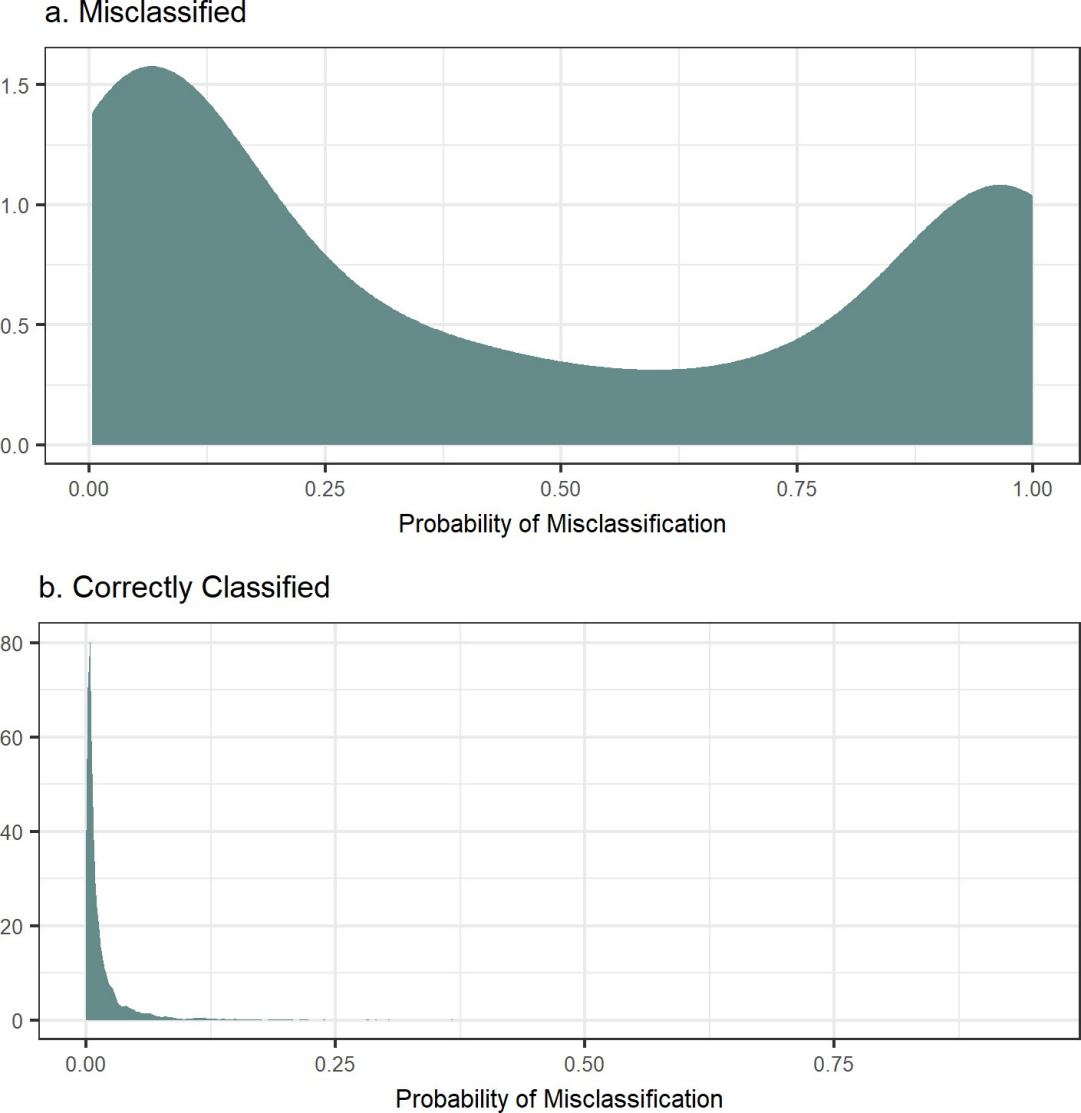
Misclassification probability densities for the a. miscoded observations and b. correctly coded observations.

In order to evaluate the ability of the proposed method to correctly identify misclassified observations, we calculated the posterior probability of misclassification of each observation in the data set. The average misclassification probability of miscoded observations was over twenty times higher than that of the non-miscoded observations. For the 95% truly non-misclassified and 5% misclassified observations, their average misclassification probability was 0.026 and 0.554, respectively. Fig 1 presents the distribution of misclassification probability for the miscoded observations (Fig 1A) and the correctly coded observations (Fig 1B) for one replicate. The 85^th^ percentile of correctly classified observations was 0.02613, while the 15^th^ percentile of misclassified observations was 0.0297. This is important as it shows that the algorithm was able to distinguish between the two groups and the miscoded records were detected with a high probability.

The correlation between the true and estimated breeding values under the different models for the symmetric scenario is presented in Table 4. As expected, the highest correlation (0.378) was obtained when the data was free of misclassification (M1), and the minimum (0.300) when the data was noisy and misclassification was ignored during the analysis (M2). Using the noisy data and the proposed methods, the correlation was 0.350 and 0.348 for M3 and M4, respectively. Under the null model (M5), the correlation was the same as in M1. Under the non-symmetric simulation scenario, the correlation between true and estimated breeding values was equal to 0.36 and 0.356 for D1 and D2, respectively.

**Table 4:** Pearson correlation between true and estimated breeding values under different models^1^ and datasets^2^.

	D1	D2
M1	0.378	0.370
M2	0.300	0.314
M3	0.350	0.346
M4	0.348	0.347
M5	0.377	0.370

^1^ M1: True data analysis with a classical threshold model, M2: Noisy data analyzed with a classical threshold model, M3: noisy data analyzed with the proposed method assuming the misclassification probability is known, M4: same as M3 except the misclassification probability is assumed unknown, M5: Noise free data analyzed using our proposed method (null model);

^2^ D1: large dataset, D2: small dataset

For the small simulated data, due to the fact that the true parameters of the model were estimated with large uncertainty, the performance of the different methods was compared to M1 as reference. This choice is motivated by the fact that results obtained using M1 (noise-free data and classical threshold model) are the best we can expect from the analysis of the small simulated data. Under the symmetric simulation scenario, methods M2, M4, and M5 underestimated the genetic variance by 32, 21, and 20% compared to M1, respectively, and M3 resulted in an overestimation of 12%. Although the proposed methods (M3, M4) for dealing with misclassification were unable to reproduce the results obtained using the noise-free data (M1), they reduced the bias by more than half compared to M2. For M4, misclassification probabilities were also underestimated with posterior means equal to 0.013, 0.007, and 0.007 for π_12_, *π*_23_, and *π*_13_, respectively. Correlations between true and predicted breeding values (Table 4) followed the same trend as those for the large data set, although they were a bit smaller in magnitude.

The real data was analyzed assuming nonsymmetric misclassification probabilities between categories. The estimated genetic variance was 0.326 and 0.118 using a classical threshold model (M1) and our proposed method, respectively. Although there is no true value to compare with, estimates obtained using our method are more realistic for a trait characterized by low heritability [40–41]. Misclassification probabilities were around zero (0.001) for *π*_31_ and *π*_13_, and approximately ten-fold higher (0.0098–0.0103) for *π*_12_, *π*_21_, *π*_23_, and *π*_32_. These results are expected given the unlikely misclassification between the third category (major calving difficulty) and the other two categories and any potential misclassification is likely the result of a posterior assessment of the calving event.

## Discussions

It has been well-established in the literature that misclassification in the response variable, especially discrete responses, will lead to biased estimation of parameters in the model [1]. The results of this study showed clearly that a small noise level (5%) in an ordered categorical response (3 categories) resulted in a substantial bias in the estimation of genetic variance and breeding values. In animal and plant improvement programs such inaccurate estimates will negatively impact the efficiency of selection.

**Fig 2:**
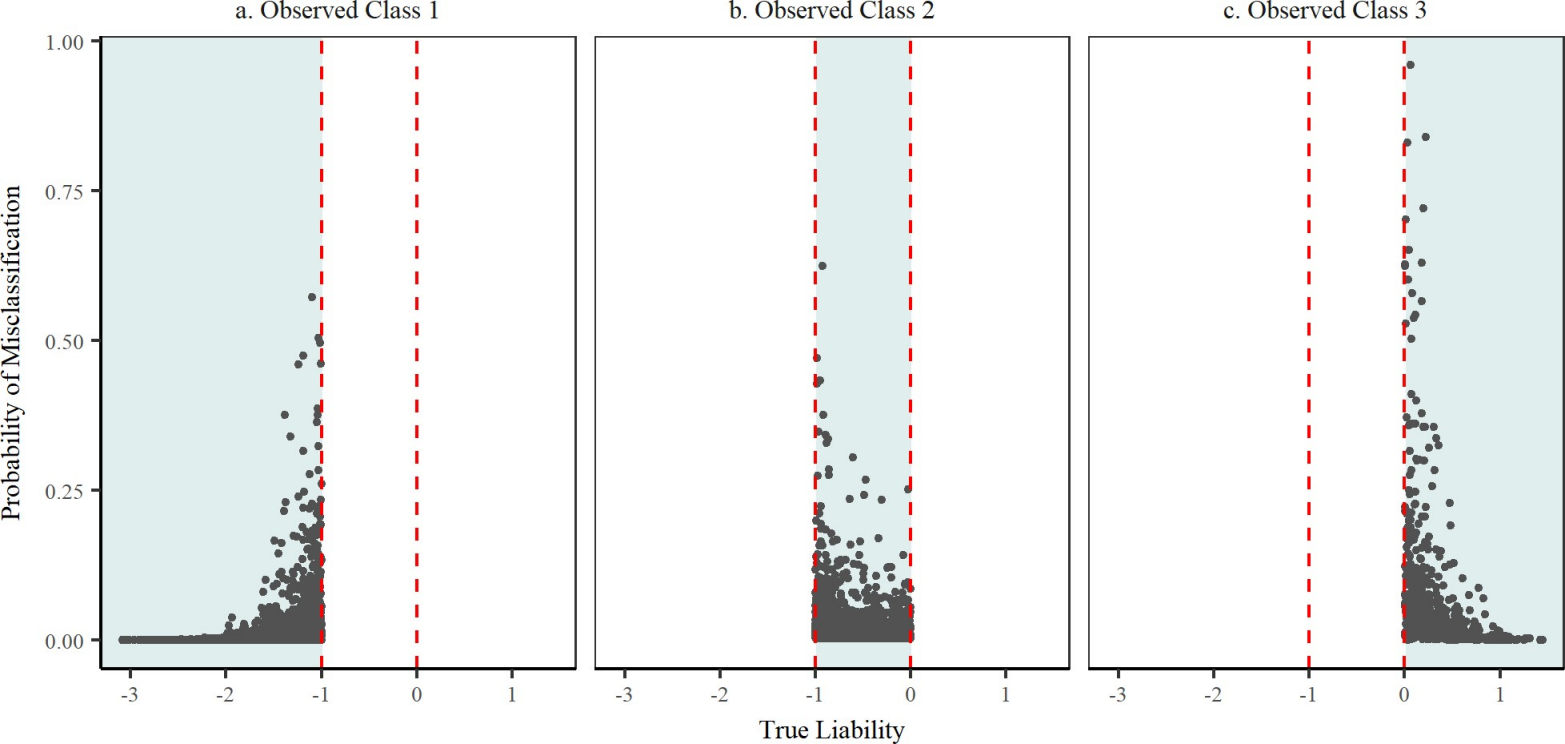
Probability of misclassification of correctly classified observations. Dashed red lines indicate the threshold values separating the three categories.

**Fig 3:**
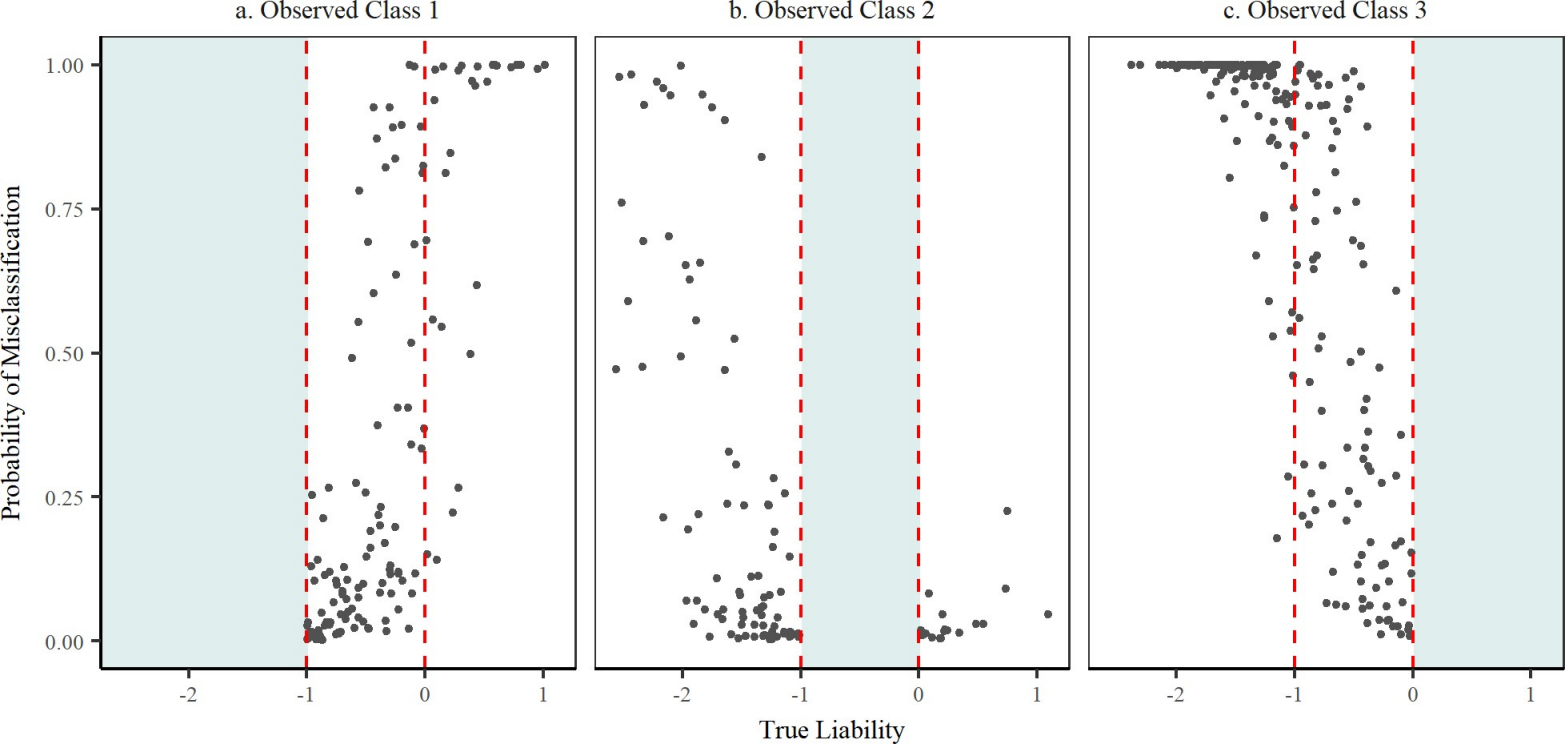
Probability of misclassification of miscoded observations. Dashed red lines indicate the threshold values separating the three categories. Individual plots are separated by the observed (miscoded) class of observations. Observations are plotted along the x-axis by their true liability, indicating their true class.

The methodology discussed here is an extension to the work carried out by our group on the analysis of binary data subject to misclassification [5–6,11]. It proposes to address misclassification by identifying individual observations that are miscoded followed by switching (reassignment) to their true class. In doing so, a ‘true’ dataset is produced from which estimates of parameters are inferred. While it is unlikely that all misclassified observations will be successfully detected in any practical application, the results presented here show that bias can be significantly reduced by the proposed method. When the rate of misclassification was assumed known, as may be the case in some diagnostic applications, bias in estimates of genetic variance was nearly eliminated and the resulting posterior mean (0.112) was very similar to the estimate obtained for the true large sample data (Table 2). In most applications there may be reason to suspect misclassification in the data, although its extent may not be known. Even in such a scenario, the proposed method was able to substantially reduce the bias in the estimation of the genetic variance (M4; Table 2). Both when the misclassification probabilities were known or unknown, estimates of the breeding values were virtually identical to those obtained using the true noise-free data set. This is crucial for phenotype prediction and efficiency of selection. When the data was small, the proposed method was not able to completely eliminate the bias in the estimation of the genetic variance, but it helped reduce it by almost a half. This could be due to the complexity of estimating the genetic variance with small data even in the absence of misclassification. Additionally, with a small data set and a low noise level (5%) the number of misclassified observations is limited (in our case an average of 7 misclassified observations between each pair of categories), which complicates the learning of the true data generating process needed to identify potentially misclassified responses. However, the breeding values were estimated with an accuracy similar to the large data set (Table 4). For the large data set, the probability of misclassification between classes 1 and 3 (*π*_13_) was accurately estimated. However, the probability of misclassification between classes 1 and 2 (*π*_12_) and 2 and 3 (*π*_23_) were underestimated. This is not surprising due to the marked differences in liabilities between observations in more distant categories. Some observations in adjacent categories tend to have very similar liabilities (close to the separation threshold), which enormously complicates the detection of misclassified observations and facilitates the switching of truly classified responses between categories. This behavior is illustrated in Fig 2, where the probability of misclassification for correctly classified observations is highest when the true liability is located near a threshold value separating two classes. In the case of misclassified observations, the probability of misclassification is highest (almost 1) when non-adjacent categories are involved (i.e., classes 1 and 3) as indicated in Fig 3. However, when the misclassification occurs between adjacent classes, the misclassification probability is lower, especially when the liability of the switched observation is closer to the threshold separating its true and observed classes (Fig 3).

Results of the analysis of the true dataset indicate the possibility of a low level of misclassification. This is supported by the significant change in the estimation of the genetic variance when misclassification was contemplated in the model. Furthermore, estimates of the misclassification probabilities are well supported by husbandry practices. In practice, it is unlikely that a misclassification could occur between category 3 (difficult calving) and the other two categories (1 and 2), but owevemisclassification is likely between classes 1 and 2, which are more subjective.

In real applications, there is no absolute certainty about the presence of misclassification in a given data set. Thus, we tested our proposed methodology under a null model (no misclassification in the data). Under small and large data set scenarios, the estimated genetic variance and breeding values were virtually the same as when the true data was analyzed using a classical threshold model (Tables 2 and 4). Additionally, the probabilities of misclassification were small to negligible, indicating the absence of misclassification as expected.

In this study, the proposed methodology was tested using simulated datasets for an ordered categorical response with three categories and a heritability of 0.10. We assumed that the misclassification probabilities were symmetric between adjacent categories in order to facilitate the implementation. While the extension to traits with more categories and asymmetric misclassification between adjacent classes is straightforward, it could be computationally costly. For traits with higher heritability, and therefore higher predictability, performance of the methodology would be expected to improve.

## Conclusions

Large data sets are being collected in several fields of research from human heath to precision agriculture. Although this data will undoubtedly contribute towards answering complex scientific questions, the unavoidable noise, including the misclassification of discrete response variables, may result in biased inference, loss of statistical power and ultimately a less efficient decision-making process. The methodology proposed in this study provides an effective tool to at least reduce the negative impact of misclassification through the identification of potentially miscoded observations and the prediction of their true response class. Furthermore, it has been shown that the method is able to yield a similar reduction in bias whether the rate of misclassification in the data is known or to be estimated. As misclassification is seldom known before the analysis, any proposed method to deal with misclassification has to perform well in the presence of noise-free data. As indicated by the results of the null model, our method was adequate. While the application demonstrated in the current study is on simulated beef cattle production data, the methodology is flexible and would be adaptable to any ordinal response.
